# It Is a Catch-22 Situation! A Decade of Research Trends on Gay Wellbeing in China: A Bibliometric Analysis

**DOI:** 10.3390/bs15010099

**Published:** 2025-01-20

**Authors:** Jiankun Gong, Weishan Miao

**Affiliations:** 1 School of Fashion Media, Jiangxi Institute of Fashion Technology, Nanchang 330201, China; gongjiankun0@jift.edu.cn; 2 School of Journalism, Renmin University of China, Beijing 100872, China

**Keywords:** gay wellbeing, men who have sex with men (MSM) China, bibliometric analysis, LGBTQ+ health, research trends, VOS viewer

## Abstract

The wellbeing of gay men in China is shaped by a combination of cultural, social, and political factors, yet research on this topic remains fragmented despite growing global interest in LGBTQ+ health. This study provides a comprehensive bibliometric analysis of research trends on wellbeing of gays (MSM) in China, using the PRISMA 2020 guidelines for a systematic search strategy and VOSviewer for bibliometric mapping. Data from Scopus were analyzed to examine publication output, top journals, and authors, along with a co-occurrence analysis of keywords and co-authorship networks across countries and authors. Results show a steady rise in publications over the past decade, particularly after 2015, revealing strong domestic collaboration and emerging international partnerships. The study identifies key contributing journals and themes, while revealing that most research remains narrowly focused on HIV/AIDS, leaving gaps in understanding gay wellbeing from broader societal and cultural perspectives. Surprisingly, very few studies specifically examine or measure overall wellbeing, whether using quantitative or qualitative approaches, and there is limited exploration of how cultural factors influence the experiences of gay men in China. These findings underscore the need for more holistic research approaches that move beyond public health and HIV prevention to address the psychological, social, and cultural dimensions of wellbeing.

## 1. Introduction

The psychological and physical wellbeing of sexual and gender minorities has become increasingly important globally (Bhugra et al., 2022). In China, this focus is particularly crucial as gay men navigate unique challenges shaped by cultural norms, social expectations, and limited legal protections (Sun et al., 2020). Recent research highlights how traditional values and rapid social change create distinct pressures for Chinese gay men, affecting their mental health, social relationships, and overall wellbeing (Sun et al., 2020).

Specifically, the cultural and social landscape of China plays a significant role in shaping the experiences of gay men. In a society where filial piety and family lineage are highly valued, gay men often face intense pressure to marry and produce children, leading to conflicts between their sexual identity and societal expectations (Sun et al., 2020).

In China’s collectivistic society, where the needs of the family and the community often take precedence over individual desires (Chia, 2019; Longarino, 2022), gay men frequently face unique psychological challenges in upholding family expectations. Research has documented significant associations between these cultural pressures and adverse mental health outcomes (J. Li et al., 2017; Xu et al., 2017). Studies show that gay men in China experience high levels of guilt, shame, and fear of rejection, with (J. Li et al., 2017) finding that self-stigma and social pressures significantly predict depressive symptoms. The pressure to fulfill traditional roles while concealing one’s sexual identity has been linked to severe psychological consequences, with (T. Wang et al., 2018) reporting higher prevalence of depression among Chinese MSM compared to the general population. Wu et al., (2015) further documented concerning rates of suicidal ideation in this population, directly linking it to social pressures and identity concealment. These culturally specific dynamics are particularly important to highlight because they represent unique stressors within China’s social context, as demonstrated by (Xu et al., 2017), who found that internalized homophobia and mental health challenges were significantly influenced by traditional cultural values and family obligations. This differs significantly from the experiences of LGBTQ+ individuals in more progressive societies or even other sexual minorities within China.

The psychological consequences of navigating these cultural pressures are significant. T. Wang et al. (2018) report a higher prevalence of depression among Chinese MSM compared to the general population, while Wu et al. (2015) document concerning rates of suicidal ideation linked to social pressures and identity concealment. These impacts are uniquely shaped by China’s social context, with Xu et al. (2017) demonstrating how internalized homophobia and mental health challenges are significantly influenced by traditional cultural values. Catch-22 framing is crucial for understanding the fundamental dilemma faced by gay men in China. This concept specifically captures the paradoxical situation where societal structures create a double bind, empirically demonstrated through multiple studies. J. Li et al. (2017) and T. Wang et al. (2018) document significant mental health impacts regardless of whether individuals choose to disclose or conceal their identity. While individuals do exercise agency in navigating these pressures (Steward et al., 2013), the underlying structural contradiction—between filial obligations and personal authenticity—remains a Catch-22.

This tension between personal identity and societal obligations creates a Catch-22 situation for many gay men in China (Hu & Wang, 2013; Hua et al., 2019). Whether choosing to adhere to traditional expectations or pursue personal authenticity, individuals face significant psychological challenges (J. Li et al., 2017; T. Wang et al., 2018). While gay men develop various strategies to navigate these pressures, the fundamental tension between societal obligations and personal identity remains a defining feature of their experience (Sun et al., 2021).

Current research on gay men in China has predominantly focused on HIV/AIDS prevention, particularly among men who have sex with men (MSM). While this health focus is important, it overshadows other crucial aspects of wellbeing, such as mental health, identity formation, social integration, and access to broader healthcare services (Liu et al., 2022). This bibliometric analysis aims to address these gaps by mapping the current state of research on wellbeing of gay men (MSM) in China. By examining publication trends, key authors, influential papers, and emerging themes, we seek to provide a holistic view of the field’s development and identify areas requiring further investigation.

By conducting a bibliometric analysis of research trends on gay/MSM specifically, this study aims to broaden the understanding of health beyond HIV and contribute to a more nuanced discussion of gay men’s experiences in a collectivistic society. This approach will contribute to a broader understanding of the challenges faced by gay men in navigating their identities within a collectivistic society, moving beyond the traditional focus on HIV/AIDS to encompass a more holistic view of wellbeing.

Focusing only on gay men rather than the whole LGBTQ+ in China is also important due to the visibility paradox they face. On one hand, the relative visibility of gay men within the LGBTQ+ community—due to factors like media representation and the focus on MSM in public health—means that they are often perceived as the dominant or most “visible” subgroup. On the other hand, this visibility can also lead to assumptions that their challenges are well understood, overshadowing the need for in-depth research on other dimensions of their wellbeing. This paradox creates a Catch-22 situation: while gay men may be visible in certain aspects, their broader psychological and social health needs are often neglected in both research and practice.

In line with the above discussion, this study mainly addresses the following objectives:

1. To analyze the temporal trends in publication output on gay/MSM wellbeing in China, identifying patterns of growth or decline in research interest over time.
2. To identify the top journals and authors contributing to the field of gay/MSM wellbeing research in China, highlighting the key platforms and researchers driving the discourse.
3. To conduct a co-occurrence analysis of keywords to map the main themes and concepts within the research landscape of gay/MSM wellbeing in China, uncovering potential research gaps and emerging areas of focus.
4. To perform a co-authorship analysis of authors and countries, revealing collaboration patterns and international research networks in the field of gay/MSM wellbeing in China.
5. To identify potential gaps in the existing literature and suggest future research directions that could contribute to a more holistic understanding of gay/MSM wellbeing in the Chinese context.

## 2. Methodology

The methodology of this research employs bibliometric analysis to systematically explore the existing literature on gay wellbeing in China. The study begins with data collection from the Scopus database, chosen for its extensive coverage of peer-reviewed literature across various disciplines, which is crucial for ensuring a comprehensive understanding of the topic (Sikandar et al., 2023). A well-defined search strategy is implemented, utilizing a targeted search string that includes keywords related to gay wellbeing in China while filtering results to encompass publications from 2013 to 2024 (the last decade). The search string was (“gay men” OR “homosexual men” OR “MSM” OR “men who have sex with men” OR “sexual minorities” OR “gay wellbeing” OR “gay health”) AND (“wellbeing” OR “health” OR “mental health” OR “physical health” OR “psychological health” OR “psychological wellbeing” OR “Subjective wellbeing” OR “social support” OR “discrimination” OR “stigma” OR “family pressure” OR “sexual health”) AND (“China” OR “Chinese” OR “PRC” OR “People’s Republic of China”) This rigorous approach initially yields 1356 results, from which 953 relevant articles are selected based on predefined inclusion criteria. This study specifically focuses on the experiences of gay men and men who have sex with men (MSM) in China, rather than addressing the broader LGBTQ+ spectrum. The targeted focus was intentionally designed to avoid homogenizing the diverse experiences within the LGBTQ+ community and to provide a deeper understanding of the unique challenges faced by gay men in the Chinese cultural context. The terminology used in this study reflects the existing academic discourse in China. The term “queer”, while recognized internationally, is less commonly utilized in Chinese academic literature on this topic. Our search strategy and inclusion criteria were specifically designed to capture research that directly addresses the wellbeing of gay men and MSM in China. Expanding the scope to include broader terms such as “queer” or “sexual diversities” might have diluted the focused examination of the issues unique to this subgroup, thereby diverging from the study’s primary objective.

Following data collection, the analysis phase employs VOS viewer 1.6 software, a prominent tool for constructing and visualizing bibliometric networks (Van Eck & Waltman, 2014). The analysis encompasses several components: co-occurrence analysis of keywords aims to identify major research themes and their interrelations through network diagrams; co-authorship analysis examines collaboration patterns among countries and authors, visualizing these relationships to highlight key researchers and collaborative networks (Vaicondam et al., 2022), ultimately creating a network diagram that illustrates citation relationships among key documents.

**Figure 1:**
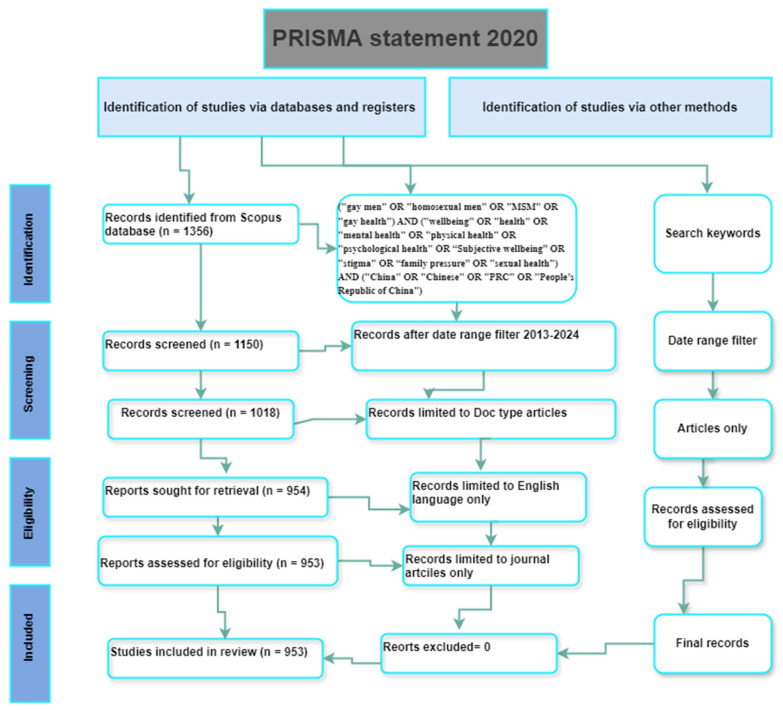
Prisma statement 2020 for inclusion and exclusion of articles.

Data interpretation further enriches the findings through thematic analysis of keyword clusters to uncover significant research themes and gaps, as well as an examination of co-authorship networks to elucidate the structure of research collaboration (Mustapha et al., 2023). The impact of the research is assessed by identifying highly cited documents and their contributions to the field. This methodological framework adheres to the PRISMA 2020 guidelines (as shown in Figure 1), ensuring a rigorous approach to systematic review and meta-analysis in bibliometric studies (Moher et al., 2015). By integrating these methodologies, this study aims to provide a comprehensive overview of gay/MSM wellbeing research in China, contributing valuable insights into both its current state and future directions.

## 3. Descriptive Statistics

### 3.1. Year-Wise Classification

**Figure 2:**
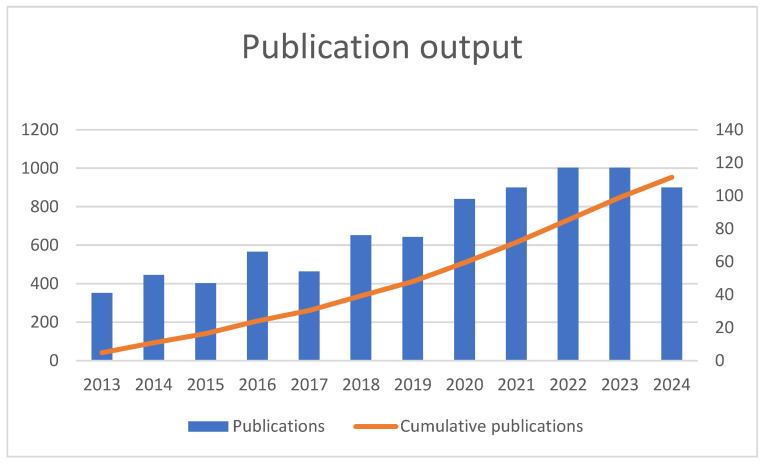
Year-wise classification of publications.

Figure 2 presents data on the number of publications produced annually from 2013 to 2024, alongside their cumulative totals. In 2013, 41 publications were recorded, and this number steadily increased in the following years. By 2014, the total publications reached 93, reflecting the addition of 52 new works. This trend continued, with notable growth from 2018 to 2022, where the annual publications jumped from 76 in 2018 to 117 in both 2022 and 2023. The cumulative total consistently rose, surpassing 500 publications by 2020 and reaching 731 by the end of 2022. Despite a slight dip in 2024, with 105 publications compared to 117 in the previous two years, the cumulative number reached an impressive total of 953 by the end of 2024. These data highlight a consistent and sustained increase in publication activity over the 12-year period.

### 3.2. Top Authors

Table 1 presents the top ten authors in research on gay men’s wellbeing, ranked by the number of publications. Tang Weiming from Southern Medical University, China, leads with 102 publications, 5628 citations, and an H-index of 37, indicating his high productivity and impact. Joseph D. Tucker from the University of North Carolina at Chapel Hill, U.S., follows with 78 publications, 5131 citations, and an H-index of 35. Chongyi Wei from Rutgers School of Public Health, U.S., has 47 publications and 3832 citations, with an H-index of 35. Other notable contributors include Jason J. Ong (Monash University, Australia), Joseph T.F. Lau (Chinese University of Hong Kong, with a remarkable 127,630 citations), and several researchers from China, Hong Kong, and the United Kingdom. These authors have made substantial contributions to the field, with high citation counts reflecting the influence of their work on the research community.

### 3.3. Top Journals

**Figure 3:**
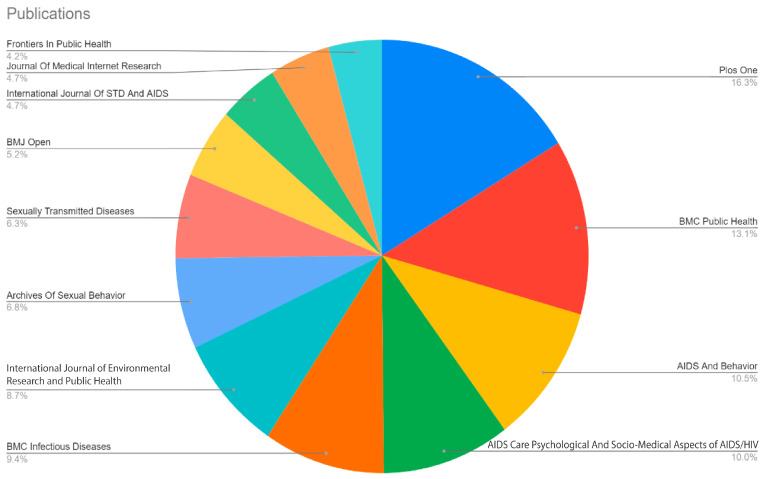
Top journals in the field of gay wellbeing in China.

We have identified the top journals that published most of the relavant papers in the field. Figure 3 highlights the top journals with more than 15 publications focused on research related to gay men’s wellbeing. *Plos One* stands out with 62 publications, making it the most prolific in this field. *BMC Public Health* follows closely with 50 publications, indicating its significant contribution to the discourse. *AIDS And Behavior* and *AIDS Care* contribute 40 and 38 publications, respectively, while *BMC Infectious Diseases* adds 36. *The International Journal of Environmental Research and Public Health* accounts for 33 publications. Other journals with notable contributions include *Archives of Sexual Behavior* (26), *Sexually Transmitted Diseases* (24), *BMJ Open* (20), *International Journal of STD and AIDS* (18), *Journal of Medical Internet Research* (18), and *Frontiers in Public Health* (16).

### 3.4. Top Cited Papers

This Table 2 highlights the top-cited papers in research related to gay men’s wellbeing in China. Zou and Fan’s (2017) paper, focusing on the use of smartphone geosocial networking applications and their implications for HIV interventions, leads with 126 citations. A 2014 study by Wei et al. (2014) on accessing HIV testing and treatment in China follows with 110 citations, while W. Tang et al.’s (2018) work on expanding HIV testing through crowdsourcing ranks third with 108 citations. Other notable papers address topics such as gay apps for sexual health, internalized homophobia, and circumcision as a preventive measure for HIV. These highly cited works reflect significant contributions to understanding the health and social challenges faced by gay men in China, with a focus on HIV prevention, mental health, and the role of technology.

## 4. Bibliometric Analysis

### 4.1. Co-Authorship of Authors

The co-authorship analysis presented in this section aims to identify the most influential and collaborative authors in the research area under study. The analysis was conducted using VOS viewer. To ensure a focused analysis, the study set a minimum threshold of five documents per author and five citations. This filtering criterion resulted in the inclusion of 304 authors out of the initial 3415 authors in the dataset. In co-authorship analysis, “Links” represents the number of unique co-authors, “Total link strength (TLS)” indicates the intensity of collaborations, “Documents” shows an author’s publication count, and “Citations” reflects the impact of an author’s work in the field (as shown in Table 3).

The co-authorship analysis of authors researching wellbeing of gays (MSM) in China, as visualized in the VOS viewer network map and supported by the accompanying data table, provides valuable insights into the collaborative landscape of this field.

**Figure 4:**
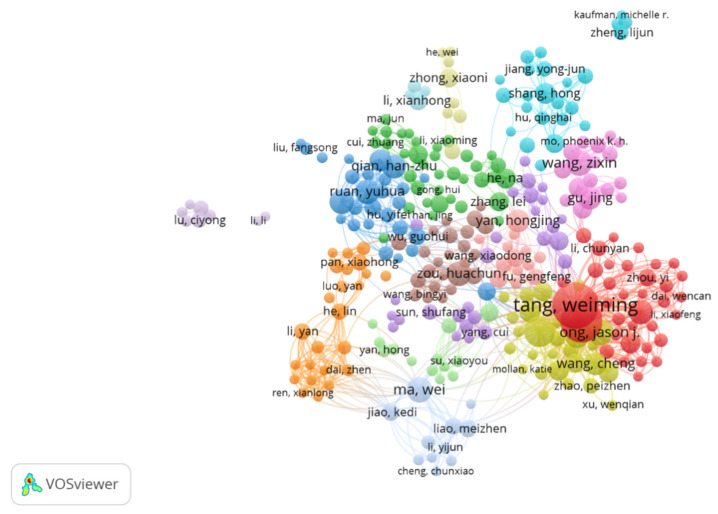
Snapshot of co-authorship of authors analysis. Available online at https://bit.ly/3C0A7vy, accessed on 10 September 2024.

The network visualization reveals a complex web of collaborations, with authors grouped into distinct clusters represented by different colors. At the center of this network, we find several prominent researchers who appear to be key players in the field (Figure 4).

Tang Weiming emerges as the most prolific and influential author in this research domain. With the highest number of documents (99), citations (1550), and total link strength (695), Tang’s central position in the yellow cluster (Cluster 1) indicates a pivotal role in shaping the research landscape. Closely connected to Tang is Tucker Joseph D., also in Cluster 1, with 84 documents and 1275 citations, suggesting a strong collaborative relationship between these two researchers.

Other significant contributors include Wei Chongyi (Cluster 4, 46 documents, 1226 citations) and Ong Jason J. (Cluster 4, 32 documents, 275 citations), who form another important collaborative group. Their high citation counts relative to document numbers suggest that their work has considerable impact in the field.

The network also highlights the importance of Chinese researchers in this domain, which is expected given the focus on gay wellbeing in China. Authors like Ma Wei (Cluster 12, 30 documents, 299 citations), Ruan Yuhua (Cluster 3, 29 documents, 662 citations), and Zou Huachun (Cluster 8, 29 documents, 511 citations) represent different research clusters, indicating diverse approaches or focus areas within the broader topic. Interestingly, some authors like Shao Yiming (Cluster 3, 29 documents, 630 citations) and Yan Hongjing (Cluster 10, 24 documents, 620 citations) have relatively high citation counts compared to their number of documents, suggesting that their work, while less prolific, has a significant impact in the field.

This co-authorship analysis not only identifies key researchers but also illuminates the collaborative nature of research in this field. The intricate connections between authors across different clusters indicate a high degree of interdisciplinary and inter-institutional collaboration, which is crucial for addressing complex social and health issues (Katz & Martin, 1997).

In conclusion, this analysis provides a comprehensive overview of the key contributors and collaborative networks in research on gay wellbeing in China. It highlights the central roles of certain researchers, the diversity of research clusters, and the interconnected nature of scholarship in this field. This information can be valuable for identifying potential collaborators, understanding the structure of the research community, and recognizing influential work in the domain of gay wellbeing in China.

### 4.2. Co-Authorship of Countries

The analysis of co-authorship among countries offers important insights into global collaboration trends and the research dynamics within the studied field. For this analysis, a minimum of two publications per country and no citation requirement were applied, which led to the selection of 24 countries from a total of 45 in the dataset (as shown in Table 4).

The co-authorship network of countries (Table 2) clearly demonstrates China’s dominant position in research related to gay wellbeing in China, which is entirely expected given that China was a key search term. China stands at the center of this research landscape with the highest number of documents (813), citations (11,254), and total link strength (682). This overwhelming lead is natural, as the study specifically focused on gay wellbeing in China.

The United States emerges as the second most significant contributor, with 378 documents and 6778 citations, indicating strong international collaboration with Chinese researchers. This collaboration is likely due to the US’s prominent role in global health and social science research.

Hong Kong and Macao, while administratively part of China, are represented separately in this analysis. Hong Kong shows substantial involvement with 147 documents and 1635 citations, reflecting its role as a major research hub and its unique position bridging Chinese and international academic communities. Macao’s contribution is smaller but still noteworthy.

Other major contributors include the United Kingdom (103 documents, 1327 citations) and Australia (98 documents, 1380 citations), highlighting the global interest in this topic. These countries, along with Canada, form a cluster of English-speaking nations with strong research ties to China in this field.

Asian countries like Japan, Singapore, Taiwan, and Thailand also appear in the network, suggesting regional interest and collaboration. European nations such as Switzerland, the Netherlands, and Sweden contribute to the research, albeit with fewer documents.

Interestingly, some countries with relatively few documents show high citation counts or link strengths, indicating influential contributions. For example, Malawi has only three documents but 99 citations, suggesting impactful research despite limited output.

**Figure 5:**
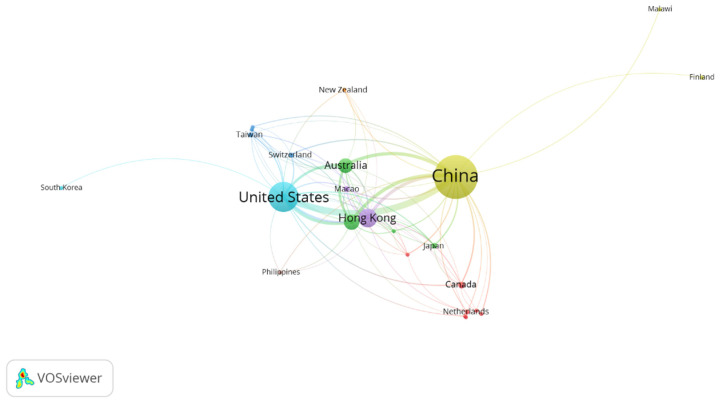
Screenshot of co-authorship analysis of countries in network visualization mode; available online at https://bit.ly/4fbxQfr, accessed on 10 September 2024.

This network (Figure 5) clearly illustrates that while research on gay wellbeing in China is primarily conducted within China, it has attracted significant international attention and collaboration. The global nature of this research field is evident, with contributions spanning multiple continents and forming various collaborative clusters, all centering around and connecting back to China as the primary focus of the studies.

Figure 5 visually represents the co-authorship network of countries researching gay wellbeing in China. China appears as the largest node at the center, reflecting its dominant role as both the subject of study and primary contributor. The network is organized into several distinct clusters, each represented by a different color. The United States forms another large node, closely linked to China, indicating strong bilateral collaboration. Hong Kong and Macao are positioned near China, forming a cluster that likely represents research within Greater China. Australia and the United Kingdom form another significant cluster, suggesting a commonwealth or English-speaking research community. Asian countries like Japan, Taiwan, and Singapore create a regional cluster, while European nations form a more loosely connected group. The size of each node corresponds to the country’s research output, while the thickness of the lines between nodes represents the strength of collaboration. Countries like Finland and Malawi appear as smaller, peripheral nodes, indicating more limited but still present international reach in this research field. Overall, the figure effectively illustrates the global nature of research on gay wellbeing in China, with clear patterns of regional and linguistic collaboration, all centering around China as the focal point.

### 4.3. Co-Occurrence of Keywords

We performed a co-occurrence analysis of author-provided keywords to uncover the main research themes and concepts and analyze the trending and least-explored keywords related to the field. The analysis began by setting a minimum occurrence threshold of five, which reduced the keywords to 98 from a total of 1668 in Table 5. After consolidating similar keywords, the final count was refined to 62 from the initial 1630.

The co-occurrence analysis of keywords reveals significant trends and focal points in the field. The most prominent keyword is “gay men” with the highest total link strength (722) and occurrences (346), indicating its central role in the research landscape. This is because the similar keywords, such as MSM, homosexual men, men who have sex with men, Chinese gay men, gay, gay and bisexual men, homosexuality, men who have sex with men (MSM), young men who have sex with men, were all merged into one common term, which is gay men, for the purpose of the co-occurrence of keyword analysis. This is closely followed by “China” and “HIV/AIDS”, both with high link strengths and occurrences, underscoring the geographical focus of the studies and the predominant health concern addressed in this research area.

**Figure 6:**
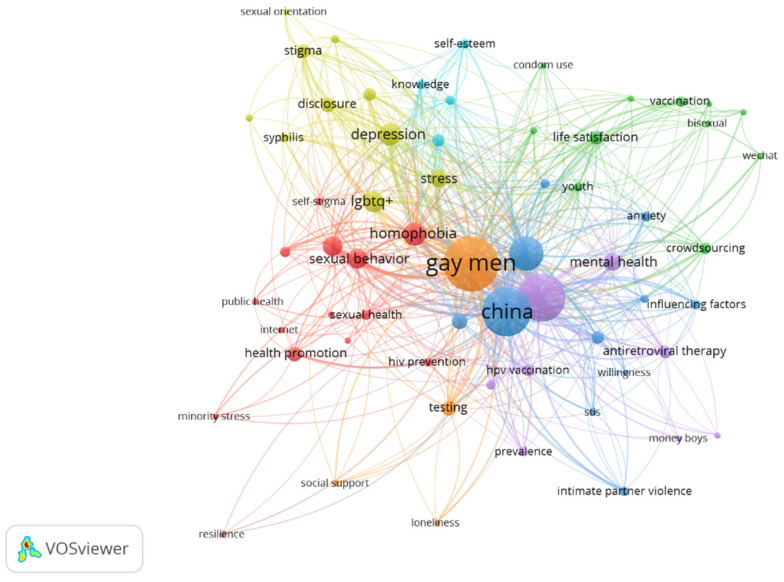
Screenshot of the co-occurrence of keyword analysis; available online at https://bit.ly/3Uc1lpy, accessed on 10 September 2024.

The keyword “PrEP” (Pre-Exposure Prophylaxis) emerges as a significant topic with a high link strength (307) suggesting a growing focus on HIV prevention strategies. Mental health-related keywords such as “depression”, “stress”, and “anxiety” also feature prominently, indicating an increasing emphasis on psychological wellbeing alongside physical health concerns in Figure 6.

Social and cultural aspects of gay life in China are represented by keywords like “homophobia”, “stigma”, and “disclosure”, reflecting the complex societal challenges faced by this community. The presence of “migration” as a keyword with a recent average publication year (2020.783) suggests emerging research interest in the experiences of gay men who move within or outside of China.

Keywords related to HIV prevention and treatment, such as “self-testing”, “antiretroviral therapy”, and “unprotected anal intercourse”, demonstrate the continued importance of HIV/AIDS research in this field. The appearance of “crowdsourcing” with the most recent average publication year (2022.353) indicates innovative approaches being explored in research or intervention strategies.

The keyword “LGBTQ+” with a recent average publication year (2021.118) suggests a broadening of research scope to include a wider spectrum of sexual and gender identities. Keywords like “life satisfaction” and “social network” highlight the exploration of quality of life and social aspects of gay men’s experiences in China.

Overall, this co-occurrence analysis reveals a research field that is predominantly focused on health issues, particularly HIV/AIDS, but is expanding to encompass mental health, social challenges, and broader aspects of wellbeing for gay men in China. The recency of average publication years for many keywords indicates that the field is evolving, with new topics and approaches emerging in recent years. This analysis provides valuable insights into the current state and future directions of research on gay wellbeing in China, reflecting both persistent concerns and emerging areas of study.

Focusing on the aspect of gay wellbeing based on the keyword analysis, we can discern a multifaceted approach to understanding and researching the wellbeing of gay men in China. While “gay men” is the most prominent keyword, indicating the specific population focus, the co-occurrence of various other keywords paints a comprehensive picture of wellbeing research in this context.

Mental health emerges as a crucial component of gay wellbeing studies, as evidenced by the high occurrence of keywords such as “depression” (57 occurrences), “stress” (38 occurrences), and “anxiety” (14 occurrences). These keywords, all with average publication years around 2020, suggest a recent and growing emphasis on psychological aspects of gay men’s lives in China. The inclusion of “mental health” as a distinct keyword further underscores this focus as depicted in Table 6.

The presence of “life satisfaction” (23 occurrences) with an average publication year of 2020.739 indicates that researchers are exploring broader, positive aspects of wellbeing beyond just the absence of mental health issues. This aligns with contemporary approaches to wellbeing that encompass overall quality of life and subjective satisfaction.

Social dimensions of wellbeing are represented by keywords like “social network” (19 occurrences) and “disclosure” (24 occurrences). These terms suggest investigations into how social connections and the process of coming out impact the wellbeing of gay men in China. The high occurrence of “homophobia” (63 occurrences) and “stigma” (22 occurrences) points to the significant challenges that may negatively affect wellbeing in this population. While HIV/AIDS-related keywords are prominent, reflecting the historical focus on this health issue, the presence of “LGBTQ+” (51 occurrences) with a recent average publication year of 2021.118 suggests a broadening perspective that considers gay wellbeing within the larger context of sexual and gender diversity.

The keyword “influencing factors” (11 occurrences) indicates that researchers are examining the various elements that contribute to or detract from the wellbeing of gay men in China. This holistic approach is further supported by the presence of “health promotion” (24 occurrences), suggesting a proactive stance towards improving overall wellbeing. Interestingly, “migration” (46 occurrences) emerges as a significant keyword, hinting at research exploring how geographical movement, whether within China or internationally, impacts the wellbeing of gay men.

Overall, this keyword analysis reveals a research landscape that is increasingly recognizing the multidimensional nature of gay wellbeing in China. While health concerns, particularly around HIV/AIDS, remain prominent, there is a clear trend towards a more holistic understanding that encompasses mental health, social factors, life satisfaction, and the broader societal context. The recency of many of these keywords’ average publication years suggests that this more comprehensive approach to studying gay wellbeing in China is a relatively recent development, indicating an evolving and maturing field of study.

We also explored some emerging research themes by studying the keywords with low TLS and occurrences. Analyzing the least-explored keywords in research on gay wellbeing in China reveals significant opportunities for future investigation. These keywords, characterized by lower occurrences and total link strengths, indicate emerging topics that have received limited attention but are crucial for a comprehensive understanding of gay wellbeing in the Chinese context.

For instance, terms like “linkage to care” and “internet”, both appearing five times with an average publication year of 2020.8, highlight a growing interest in healthcare access and the impact of digital technologies on gay men’s lives. Future research could investigate how online platforms facilitate or obstruct access to healthcare services and support networks for gay men in China.

Other keywords such as “community engagement” and “resilience”, with average publication years of 2022 and 2020.667, respectively, underscore the importance of collective action and individual coping mechanisms. These topics could provide insights into how gay men in China foster supportive communities and develop personal strengths in adversity.

Keywords like “self-efficacy” and “sexual orientation”, despite their relevance, show low occurrences (five each). Future studies could explore how self-efficacy influences overall wellbeing and delve into the varied experiences of different sexual orientations within the broader category of gay men in China.

The inclusion of terms such as “bisexual” and “lesbian” indicates a need for more inclusive research that addresses the diverse experiences within the LGBTQ+ community in China. Focusing on these subgroups could yield valuable comparative insights and highlight unique challenges they face.

Moreover, the keyword “money boys”, referring to male sex workers, points to a specific subgroup that requires further investigation to uncover unique health risks and social challenges encountered by this vulnerable population.

The themes of “loneliness” and “social support” emphasize the critical role of social connections for wellbeing. Future research could explore interventions designed to reduce loneliness and enhance support networks for gay men in China.

Keywords like ’Money boys’ signal a need for research into vulnerable subgroups and societal pressures. The concept of “minority stress”, while underexplored, remains highly relevant. Emerging interest in “WeChat”, with an average publication year of 2022, suggests potential research into how this popular social media platform influences gay men’s lives regarding social connections and health information dissemination. Lastly, keywords like “suicidal ideation” and “willingness”, both with recent average publication years (2022), highlight critical areas for future inquiry focused on mental health risks and attitudes towards health-seeking behaviors.

By concentrating on these underexplored keywords, future researchers can address critical gaps in understanding gay wellbeing in China. This exploration may lead to more effective interventions and policies while shifting the research focus from a predominantly disease-oriented approach to a holistic understanding that encompasses social, psychological, and cultural dimensions. Overall, these keywords suggest a diversifying field that is evolving to better capture the experiences and needs of gay men within the Chinese context. Some relevant least-explored keywords are mentioned in the table below.

## 5. Discussion and Conclusions

Synthesizing findings from these highly cited papers reveals a complex landscape of challenges and opportunities for improving the wellbeing of gay men (MSM) in China. A comprehensive analysis of these studies highlights several interconnected themes.

### 5.1. HIV Prevention and Testing

Multiple studies emphasize the critical importance of innovative approaches to HIV prevention and testing among MSM in China. W. Tang et al. (2018) demonstrated the effectiveness of crowdsourcing interventions in increasing HIV testing uptake, while Zou and Fan (2017) and Bien et al. (2015) explored the potential of mobile applications and geosocial networking platforms for promoting sexual health. These findings collectively suggest that technology-driven, community-based approaches can effectively reach and engage MSM populations that may be difficult to access through traditional healthcare channels.

### 5.2. Stigma and Mental Health

A recurring theme across several studies is the significant impact of stigma on the mental health and wellbeing of MSM in China. Steward et al. (2013) and Choi et al. (2016) explored how stigma influences decision making around disclosure and coping strategies, respectively. T. Wang et al. (2018) and Wu et al. (2015) reported high prevalences of depression and suicidal ideation among HIV-positive MSM, underscoring the urgent need for mental health support in this population. These studies collectively highlight the intricate relationship between societal attitudes, individual coping mechanisms, and mental health outcomes.

### 5.3. Cultural Context and Identity

The unique cultural context of China plays a crucial role in shaping the experiences of MSM. Steward et al. (2013) examined how filial duties and cultural expectations influence decisions around marriage and disclosure. This cultural dimension adds complexity to the challenges faced by MSM in China, often requiring them to balance personal identity with societal and familial expectations.

### 5.4. Evolving Epidemic Dynamics

Zhao et al. (2016) and Y. Li et al. (2014) provided insights into the changing dynamics of the HIV epidemic among MSM in China, highlighting the importance of ongoing surveillance and adaptive prevention strategies. These studies emphasize the need for continual reassessment of HIV subtypes and transmission patterns to inform targeted interventions.

### 5.5. Pre-Exposure Prophylaxis (PrEP)

Y. Zhang et al. (2013) explored attitudes towards PrEP among MSM in Western China, finding moderate awareness but high interest in its use. This suggests potential for PrEP as a prevention strategy, but also highlights the need for education and awareness campaigns.

These studies collectively paint a picture of a population facing multiple, interrelated challenges including HIV risk, mental health issues, societal stigma, and cultural pressures. However, they also point to promising avenues for intervention, particularly through technology-driven, community-based approaches that address both physical and mental health needs. Future research and public health efforts should aim to integrate these findings, developing comprehensive strategies that consider the full spectrum of factors influencing the wellbeing of MSM in China.

This bibliometric analysis provides a comprehensive overview of the research landscape surrounding the wellbeing of MSM in China. Through the examination of co-authorship networks, keyword co-occurrences, and publication patterns, several key insights emerge.

The study reveals a vibrant and growing field of research, with China at its center both as the subject of study and the primary contributor to the literature. The significant involvement of international collaborators, particularly from the United States and other Western countries, highlights the global interest in this topic and the benefits of cross-cultural research perspectives.

The keyword analysis demonstrates that, while HIV/AIDS remains a dominant focus, the field is expanding to encompass a more holistic view of wellbeing. Mental health, social factors, and quality of life are gaining prominence, reflecting a shift towards a more comprehensive understanding of gay men’s experiences in China. This evolution suggests a maturing field that is increasingly recognizing the multifaceted nature of wellbeing beyond physical health.

However, the analysis also uncovers areas that remain underexplored, as evidenced by keywords with lower total link strengths and occurrences. These topics, often related to specific psychological, social, or technological aspects of gay men’s lives, represent potential avenues for future research. They offer opportunities to address gaps in our current understanding and contribute to a more nuanced view of gay wellbeing in the Chinese context.

The co-authorship analysis reveals a collaborative research environment, with several key researchers and institutions playing central roles. This network structure suggests a field that benefits from shared expertise and resources, while also highlighting the potential for even greater collaboration, particularly with researchers from diverse backgrounds and disciplines.

In conclusion, this bibliometric analysis paints a picture of a dynamic and evolving field of study. While significant progress has been made in understanding gay wellbeing in China, there remain ample opportunities for future research. By building on the strong foundation of existing work and exploring emerging topics, researchers can continue to contribute valuable insights that may inform policy, practice, and support for gay men in China.

### 5.6. Where Is the Way Forward?

Our analysis reveals a troubling gap in the current research on gay/MSM wellbeing in China. The majority of the literature remains predominantly focused on HIV/AIDS, with very little research addressing gay/MSM wellbeing from a broader societal perspective. Few studies have ventured beyond the realm of public health and medicine to explore the psychological, social, and cultural dimensions of wellbeing for gay men in China.

Moreover, within our systematic search focused on wellbeing, we found limited quantitative research specifically measuring wellbeing outcomes and qualitative studies examining the Catch-22 situation of gay men in China through a wellbeing lens. The focus on HIV/AIDS often overshadows the more nuanced and comprehensive aspects of wellbeing, such as mental health, societal inclusion, identity formation, and cultural influences.

Additionally, cultural factors, such as the deep-rooted concept of filial piety, are scarcely explored in the context of gay/MSM wellbeing. The pressure to adhere to traditional family roles and expectations plays a significant role in shaping the experiences of gay men in China, yet this cultural dimension remains under-researched.

Future studies should broaden the focus beyond medicalized views of gay identity to include sociocultural elements that deeply affect wellbeing. Quantitative tools specifically designed to measure overall gay/MSM wellbeing and qualitative interviews addressing cultural stressors like filial duties could provide a more holistic view of the challenges faced by this population. Furthermore, researchers should prioritize the inclusion of cultural factors in their frameworks to fully grasp how societal expectations influence gay men’s lives and mental health in China.

The way forward involves expanding the discourse to encompass the psychological, social, and cultural dimensions of wellbeing, ensuring that research on gay men in China does not remain confined to the narrow lens of HIV prevention.

## 6. Limitations

This bibliometric analysis, while comprehensive, has several limitations that should be acknowledged. The reliance on a single database (Scopus) may have excluded relevant publications not indexed therein, potentially missing important contributions. The focus on English-language publications may underrepresent research published in Chinese or other languages, limiting the analysis’s scope. While using multiple databases and including Chinese-language publications could provide additional insights, this would require different methodological approaches beyond the scope of current bibliometric analysis frameworks. The bibliometric approach provides valuable insights into research patterns; it does not assess the quality or specific findings of individual studies, offering a quantitative overview rather than a qualitative assessment of content. Lastly, the quantitative nature of bibliometric analysis may not fully capture the nuanced qualitative contributions to the field of gay/MSM wellbeing research in China. While our bibliometric analysis comprehensively covers research explicitly focused on gay/MSM wellbeing in China, we acknowledge that valuable studies exploring the social and cultural experiences of gay men in China, including their navigation of societal pressures, may exist in literature beyond our wellbeing-focused search parameters. This is particularly relevant for qualitative research addressing identity formation, community engagement, and resilience that may not explicitly use wellbeing terminology.

## Figures and Tables

**Table 1:** Top authors in the field with most publications.

Author Name	Affiliation	Country	Publications	Citations	H Index
Tang, Weiming	Southern Medical University	China	102	5628	37
Tucker, Joseph D.	The University of North Carolina at Chapel Hill	United States	78	5131	35
Wei, Chongyi	Rutgers School of Public Health	United States	47	3832	35
Ong, Jason J.	Monash University	Australia	38	4172	32
Lau, Joseph T.F.	Chinese University of Hong Kong	Hong Kong	35	127,630	137
Wang, Zixin	Chinese University of Hong Kong, Faculty of Medicine	Hong Kong	34	2207	25
Wu, Dan	London School of Hygiene and Tropical Medicine	United Kingdom	30	2367	25
Ruan, Yuhua	Chinese Center for Disease Control and Prevention	China	29	6115	40
Shao, Yiming	Changping Laboratory	China	29	12,769	50
Zou, Huachun	Fudan University	China	29	4642	34

**Table 2:** Top cited articles.

Paper Source	Journal	Cited by	Focus of the Study
(Zou & Fan, 2017)	*Archives of Sexual Behavior*	126	Characteristics of MSM using geosocial networking apps and implications for HIV interventions
(Wei et al., 2014)	*AIDS Care-Psychological and Socio-Medical Aspects of AIDS/HIV*	110	Barriers and facilitators to HIV testing and treatment among MSM in China
(W. Tang et al., 2018)	*PLoS Medicine*	108	Effectiveness of crowdsourcing to expand HIV testing among MSM in China
(Bien et al., 2015)	*AIDS and Behavior*	106	Use of gay apps for seeking sex partners in China and implications for MSM sexual health
(W. Tang et al., 2016)	*Clinical Infectious Diseases*	103	Effectiveness of crowdsourced HIV test promotion videos for MSM in China
(S. Tang et al., 2016)	*BMC Infectious Diseases*	95	HIV epidemiology and responses among MSM and transgender individuals in China
(Xu et al., 2017)	*International Journal for Equity in Health*	94	Internalized homophobia, mental health, sexual behaviors, and outness of gay/bisexual men in Southwest China
(Y. Li et al., 2014)	*AIDS*	92	Association of HIV-1 subtypes with disease progression in Chinese patients
(J. Li et al., 2017)	*AIDS and Behavior*	83	Roles of self-stigma, social support, and affects as determinants of depressive symptoms among HIV-infected MSM in China
(J. Zhang et al., 2022)	*The Lancet HIV*	81	Discontinuation, suboptimal adherence, and reinitiation of oral HIV pre-exposure prophylaxis globally
(Yuan et al., 2019)	*The Lancet Global Health*	79	Effectiveness of circumcision in preventing HIV and other STIs among MSM globally
(Z. Wang et al., 2018)	*Chinese Medical Journal*	79	Risk factors for carbapenem-resistant Klebsiella pneumoniae infection and mortality
(Steward et al., 2013)	*PLoS ONE*	79	Influence of stigma and filial duties on marital decisions among Chinese MSM
(Zhong et al., 2017)	*HIV Medicine*	77	Acceptability and feasibility of a social entrepreneurship testing model for HIV self-testing among MSM
(Nicholls et al., 2019)	*Proceedings of the National Academy of Sciences of the United States of America*	76	Determination of mammalian germ cells after PGC colonization of the nascent gonad
(Choi et al., 2016)	*Archives of Sexual Behavior*	75	Sexual stigma, coping styles, and psychological distress among MSM in Beijing, China
(T. Wang et al., 2018)	*BMC Psychiatry*	73	Prevalence of depression or depressive symptoms among people living with HIV/AIDS in China
(Wu et al., 2015)	*International Journal of STD and AIDS*	71	Prevalence of suicidal ideation and associated factors among HIV-positive MSM in Anhui, China
(Zhao et al., 2016)	*Scientific Reports*	65	Dynamics of the HIV epidemic among MSM in Shenzhen, China from 2005 to 2012
(Y. Zhang et al., 2013)	*AIDS Patient Care and STDs*	64	Attitudes toward HIV pre-exposure prophylaxis among MSM in Western China
(Pyun et al., 2014)	*Asia-Pacific Journal of Public Health*	64	Impact of internalized homophobia on HIV testing among MSM in China

**Table 3:** Top collaborating authors in the field according to their TLS.

Authors	Cluster	Links	Total Link Strength	Documents	Citations
Tang, Weiming	1	116	695	99	1550
Tucker, Joseph D.	1	98	574	84	1275
Wei, Chongyi	4	68	305	46	1226
Wu, Dan	1	54	263	29	322
Ma, Wei	12	70	241	30	299
Ong, Jason J.	4	51	226	32	275
Yang, Bin	4	64	225	28	611
Ruan, Yuhua	3	38	182	29	662
Zhang, Ye	4	57	180	21	443
Zou, Huachun	8	51	176	29	511
Huang, Wenting	1	45	171	20	240
Fu, Hongyun	4	39	169	19	254
Shao, Yiming	3	35	157	29	630
Wang, Cheng	4	46	156	24	225
Liu, Chuncheng	4	41	152	20	333
Qian, Han-Zhu	3	50	143	26	662
Cao, Bolin	4	40	142	18	311
Yan, Hongjing	10	43	133	24	620
Shang, Hong	6	30	128	21	382
Jiao, Kedi	12	27	126	14	43

**Table 4:** Top contributing countries as per their TLS.

Countries	Cluster	Links	Total Link Strength	Documents	Citations
China	4	21	682	813	11,254
United States	6	18	546	378	6778
United Kingdom	2	14	244	103	1327
Australia	2	13	201	98	1380
Hong Kong	5	15	161	147	1635
Macao	5	7	45	15	236
Switzerland	3	9	32	13	327
Japan	2	7	29	15	258
Canada	1	6	28	20	464
Singapore	2	8	21	8	32
Taiwan	3	7	18	11	231
Thailand	1	8	17	9	46
Netherlands	1	7	12	10	217
Malaysia	3	7	11	6	73
India	3	5	10	6	188
Sweden	1	5	10	5	87
Belgium	1	3	9	9	133
New Zealand	7	5	9	5	83
Philippines	8	4	8	3	40
Germany	1	5	5	2	80
Italy	1	3	3	2	38
Malawi	4	1	3	3	99
Finland	4	1	2	2	0
South Korea	6	1	2	2	8

**Table 5:** Results of the co-occurrence of keyword analysis.

Keyword	Cluster	Links	Total Link Strength	Occurrences	Avg. Pub. Year
Gay Men	7	58	722	346	2019.74
China	3	53	608	268	2019.224
HIV/AIDS	5	53	568	256	2019.43
Prep	3	52	307	137	2020.088
Depression	4	32	154	57	2020.474
Homophobia	1	37	133	63	2019.556
Migration	1	27	107	46	2020.783
Sexual Behavior	1	31	100	48	2020.354
Stress	4	24	96	38	2020.263
LGBTQ+	4	24	92	51	2021.118
Mental Health	5	25	70	38	2019.263
Self-Testing	3	19	61	29	2020.655
Stigma	4	18	59	22	2021.455
Testing	7	21	58	27	2020.926
Antiretroviral Therapy	5	18	56	20	2018.7
Disclosure	4	22	52	24	2021.833
Attitude	4	17	50	18	2020.333
Crowdsourcing	2	15	50	17	2022.353
Epidemiology	6	18	49	20	2021.25
Life Satisfaction	2	22	45	23	2020.739
Social Network	3	16	45	19	2020.737
Health Promotion	1	14	35	24	2020
Unprotected Anal Intercourse	3	16	34	9	2020.222
Anxiety	3	13	33	14	2019.357
Influencing Factors	3	11	32	11	2019.546

**Table 6:** Least-explored keywords in the field.

Keyword	Links	Total Link Strength	Occurrences	Avg. Pub. Year
Linkage To Care	5	5	5	2020.8
Internet	5	6	5	2020.8
Mpox	5	7	5	2015.8
Community Engagement	5	9	7	2022
Resilience	4	9	6	2020.667
Self-Efficacy	6	9	5	2017.2
Sexual Orientation	6	9	5	2022.2
Bisexual	9	11	5	2020.2
Money Boys	7	11	5	2020
Public Health	7	11	5	2021.8
Condom Use	10	12	5	2020
Incidence	8	12	5	2020
Lesbian	8	12	5	2020.4
Loneliness	6	12	5	2019.2
Social Support	7	12	5	2019.8
Minority Stress	6	13	5	2017.6
Wechat	8	14	5	2022
Suicidal Ideation	11	15	5	2022.8
Willingness	9	17	5	2022
HIV Prevention	12	19	9	2018.444

## References

[B1-behavsci-15-00099] Bhugra D., Killaspy H., Kar A., Levin S., Chumakov E., Rogoza D., Harvey C., Bagga H., Owino–Wamari Y., Everall I., Bishop A., Javate K. R., Westmore I., Ahuja A., Torales J., Rubin H., Castaldelli-Maia J., Ng R., Nakajima G. A., Ventriglio A. (2022). IRP commission: Sexual minorities and mental health: Global perspectives. International Review of Psychiatry.

[B2-behavsci-15-00099] Bien C. H., Best J. M., Muessig K. E., Wei C., Han L., Tucker J. D. (2015). Gay apps for seeking sex partners in China: Implications for MSM sexual health. AIDS and Behavior.

[B3-behavsci-15-00099] Chia J. L. (2019). LGBTQ rights in China: Movement-building in uncertain times. Handbook on human rights in China.

[B4-behavsci-15-00099] Choi K.-H., Steward W. T., Miège P., Hudes E., Gregorich S. E. (2016). Sexual stigma, coping styles, and psychological distress: A longitudinal study of men who have sex with men in Beijing, China. Archives of Sexual Behavior.

[B5-behavsci-15-00099] Hu X., Wang Y. (2013). LGB identity among young Chinese: The influence of traditional culture. Journal of Homosexuality.

[B6-behavsci-15-00099] Hua B., Yang V. F., Goldsen K. F. (2019). LGBT older adults at a crossroads in mainland China: The intersections of stigma, cultural values, and structural changes within a shifting context. The International Journal of Aging and Human Development.

[B7-behavsci-15-00099] Katz J. S., Martin B. R. (1997). What is research collaboration?. Research Policy.

[B8-behavsci-15-00099] Li J., Mo P. K. H., Wu A. M. S., Lau J. T. F. (2017). Roles of self-stigma, social support, and positive and negative affects as determinants of depressive symptoms among HIV infected men who have sex with men in China. AIDS and Behavior.

[B9-behavsci-15-00099] Li Y., Han Y., Xie J., Gu L., Li W., Wang H., Lv W., Song X., Li Y., Routy J.-P., Ishida T., Iwamoto A., Li T., on behalf of CACT0810 group (2014). CRF01_AE subtype is associated with X4 tropism and fast HIV progression in Chinese patients infected through sexual transmission. AIDS.

[B10-behavsci-15-00099] Liu K., Cheng F., Dong H., Dong X., Xu J. (2022). Sexual orientation and quality of life of people living with HIV/AIDS in China: Evidence from an institutional-based cross-sectional study. Quality of Life Research.

[B11-behavsci-15-00099] Longarino D. (2022). ‘Runaway legitimation’and its limits*: LGBTQ rights in China. Routledge handbook of constitutional law in greater china.

[B12-behavsci-15-00099] Moher D., Shamseer L., Clarke M., Ghersi D., Liberati A., Petticrew M., Shekelle P., Stewart L. A. (2015). Preferred reporting items for systematic review and meta-analysis protocols (PRISMA-P) 2015 statement. Systematic Reviews.

[B13-behavsci-15-00099] Mustapha I., Ali M., Khan N., Sikandar H. (2023). The impact of industry 4.0 on innovative organisations, a thematic review using the PRISMA statement 2020. International Journal of Interactive Mobile Technologies (IJIM).

[B14-behavsci-15-00099] Nicholls P. K., Schorle H., Naqvi S., Hu Y.-C., Fan Y., Carmell M. A., Dobrinski I., Watson A. L., Carlson D. F., Fahrenkrug S. C., Page D. C. (2019). Mammalian germ cells are determined after PGC colonization of the nascent gonad. Proceedings of the National Academy of Sciences.

[B15-behavsci-15-00099] Pyun T., Santos G.-M., Arreola S., Do T., Hebert P., Beck J., Makofane K., Wilson P. A., Ayala G. (2014). Internalized homophobia and reduced HIV testing among men who have sex with men in China. Asia Pacific Journal of Public Health.

[B16-behavsci-15-00099] Sikandar H., Kohar U. H. A., Corzo-Palomo E. E., Gamero-Huarcaya V. K., Ramos-Meza C. S., Shabbir M. S., Jain V. (2023). Mapping the development of open innovation research in business and management field: A bibliometric analysis. Journal of the Knowledge Economy.

[B17-behavsci-15-00099] Steward W. T., Miège P., Choi K.-H. (2013). Charting a moral life: The influence of stigma and filial duties on marital decisions among Chinese men who have sex with men. PLoS ONE.

[B18-behavsci-15-00099] Sun S., Hoyt W. T., Tarantino N., Pachankis J. E., Whiteley L., Operario D., Brown L. K. (2021). Cultural context matters: Testing the minority stress model among Chinese sexual minority men. Journal of Counseling Psychology.

[B19-behavsci-15-00099] Sun S., Pachankis J. E., Li X., Operario D. (2020). Addressing minority stress and mental health among men who have sex with men (MSM) in China. Current HIV/AIDS Reports.

[B20-behavsci-15-00099] Tang S., Tang W., Meyers K., Chan P., Chen Z., Tucker J. D. (2016). HIV epidemiology and responses among men who have sex with men and transgender individuals in China: A scoping review. BMC Infectious Diseases.

[B21-behavsci-15-00099] Tang W., Han L., Best J., Zhang Y., Mollan K., Kim J., Liu F., Hudgens M., Bayus B., Terris-Prestholt F., Galler S., Yang L., Peeling R., Volberding P., Ma B., Xu H., Yang B., Huang S., Fenton K., Tucker J. D. (2016). Crowdsourcing HIV test promotion videos: A noninferiority randomized controlled trial in China. Clinical Infectious Diseases.

[B22-behavsci-15-00099] Tang W., Wei C., Cao B., Wu D., Li K. T., Lu H., Ma W., Kang D., Li H., Liao M., Mollan K. R., Hudgens M. G., Liu C., Huang W., Liu A., Zhang Y., Smith M. K., Mitchell K. M., Ong J. J., Tucker J. D. (2018). Crowdsourcing to expand HIV testing among men who have sex with men in China: A closed cohort stepped wedge cluster randomized controlled trial. PLOS Medicine.

[B23-behavsci-15-00099] Vaicondam Y., Sikandar H., Irum S., Khan N., Qureshi M. I. (2022). Research landscape of digital learning over the past 20 years: A bibliometric and visualisation analysis. International Journal of Online and Biomedical Engineering (IJOE).

[B24-behavsci-15-00099] Van Eck N. J., Waltman L., Ding Y., Rousseau R., Wolfram D. (2014). Visualizing bibliometric networks. Measuring scholarly impact.

[B25-behavsci-15-00099] Wang T., Fu H., Kaminga A. C., Li Z., Guo G., Chen L., Li Q. (2018). Prevalence of depression or depressive symptoms among people living with HIV/AIDS in China: A systematic review and meta-analysis. BMC Psychiatry.

[B26-behavsci-15-00099] Wang Z., Qin R.-R., Huang L., Sun L.-Y. (2018). Risk factors for carbapenem-resistant klebsiella pneumoniae infection and mortality of klebsiella pneumoniae infection. Chinese Medical Journal.

[B27-behavsci-15-00099] Wei C., Yan H., Yang C., Raymond H. F., Li J., Yang H., Zhao J., Huan X., Stall R. (2014). Accessing HIV testing and treatment among men who have sex with men in China: A qualitative study. AIDS Care.

[B28-behavsci-15-00099] Wu Y.-L., Yang H.-Y., Wang J., Yao H., Zhao X., Chen J., Ding X.-X., Zhang H.-B., Bi P., Sun Y.-H. (2015). Prevalence of suicidal ideation and associated factors among HIV-positive MSM in Anhui, China. International Journal of STD & AIDS.

[B29-behavsci-15-00099] Xu W., Zheng L., Xu Y., Zheng Y. (2017). Internalized homophobia, mental health, sexual behaviors, and outness of gay/bisexual men from Southwest China. International Journal for Equity in Health.

[B30-behavsci-15-00099] Yuan T., Fitzpatrick T., Ko N.-Y., Cai Y., Chen Y., Zhao J., Li L., Xu J., Gu J., Li J., Hao C., Yang Z., Cai W., Cheng C.-Y., Luo Z., Zhang K., Wu G., Meng X., Grulich A. E., Zou H. (2019). Circumcision to prevent HIV and other sexually transmitted infections in men who have sex with men: A systematic review and meta-analysis of global data. The Lancet Global Health.

[B31-behavsci-15-00099] Zhang J., Li C., Xu J., Hu Z., Rutstein S. E., Tucker J. D., Ong J. J., Jiang Y., Geng W., Wright S. T., Cohen M. S., Shang H., Tang W. (2022). Discontinuation, suboptimal adherence, and reinitiation of oral HIV pre-exposure prophylaxis: A global systematic review and meta-analysis. The Lancet HIV.

[B32-behavsci-15-00099] Zhang Y., Peng B., She Y., Liang H., Peng H.-B., Qian H.-Z., Vermund S. H., Zhong X.-N., Huang A. (2013). Attitudes toward HIV pre-exposure prophylaxis among men who have sex with men in western China. AIDS Patient Care and STDs.

[B33-behavsci-15-00099] Zhao J., Chen L., Chaillon A., Zheng C., Cai W., Yang Z., Li G., Gan Y., Wang X., Hu Y., Zhong P., Zhang C., Smith D. M. (2016). The dynamics of the HIV epidemic among men who have sex with men (MSM) from 2005 to 2012 in Shenzhen, China. Scientific Reports.

[B34-behavsci-15-00099] Zhong F., Tang W., Cheng W., Lin P., Wu Q., Cai Y., Tang S., Fan L., Zhao Y., Chen X., Mao J., Meng G., Tucker J., Xu H. (2017). Acceptability and feasibility of a social entrepreneurship testing model to promote HIV self-testing and linkage to care among men who have sex with men. HIV Medicine.

[B35-behavsci-15-00099] Zou H., Fan S. (2017). Characteristics of men who have sex with men who use smartphone geosocial networking applications and implications for HIV interventions: A systematic review and meta-analysis. Archives of Sexual Behavior.

